# Removal of Chromium Species by Adsorption: Fundamental Principles, Newly Developed Adsorbents and Future Perspectives

**DOI:** 10.3390/molecules28020639

**Published:** 2023-01-08

**Authors:** Bo Liu, Ya-Nan Xin, Jiao Zou, Fazal Muhammad Khoso, Yi-Ping Liu, Xin-Yu Jiang, Sui Peng, Jin-Gang Yu

**Affiliations:** 1 State Key Laboratory of Vanadium and Titanium Resources Comprehensive Utilization, Panzhihua 617000, China; 2 College of Chemistry and Chemical Engineering, Central South University, Changsha 410083, China; 3 School of Chemistry and Materials Engineering, Huizhou University, Huizhou 516007, China

**Keywords:** adsorption, application, aqueous, chromium, removal

## Abstract

Emerging chromium (Cr) species have attracted increasing concern. A majority of Cr species, especially hexavalent chromium (Cr(VI)), could lead to lethal effects on human beings, animals, and aquatic lives even at low concentrations. One of the conventional water-treatment methodologies, adsorption, could remove these toxic Cr species efficiently. Additionally, adsorption possesses many advantages, such as being cost-saving, easy to implement, highly efficient and facile to design. Previous research has shown that the application of different adsorbents, such as carbon nanotubes (carbon nanotubes (CNTs) and graphene oxide (GO) and its derivatives), activated carbons (ACs), biochars (BCs), metal-based composites, polymers and others, is being used for Cr species removal from contaminated water and wastewater. The research progress and application of adsorption for Cr removal in recent years are reviewed, the mechanisms of adsorption are also discussed and the development trend of Cr treatment by adsorption is proposed.

## 1. Introduction

**Figure 1:**
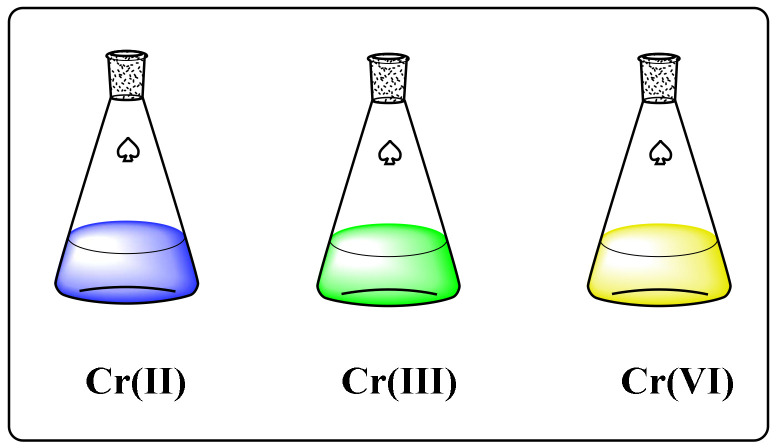
The schematic diagrams of aqueous solutions for Cr species with various oxidation states.

The oxidation of chromium (Cr) ranks from Cr(II) to Cr(VI), and Cr could exist as chromous ion [Cr(II) ion, Cr^2+^], chromic ion [Cr(III) ion, Cr^3+^], chromate ion [Cr(VI) or CrO_4_^2−^] and dichromate ion [Cr_2_O_7_^2−^] [1]. Many Cr compounds are relatively water insoluble, and Cr(II) ion is readily oxidized to Cr(III) in aqueous solutions. Therefore, in aqueous solutions, Cr would be present as Cr(III) or Cr(VI). Except for Cr(III) oxide (Cr_2_O_3_) and Cr(III) hydroxide [Cr(OH)_3_], most Cr(III) compounds are usually water insoluble because they are largely bound to the floating particles in water. As a consequence of the abovementioned physicochemical properties of Cr, the existence of three forms, namely Cr_2_O_3_, Cr(OH)_3_ and Cr(VI), in aqueous solutions has been reported and detected (Figure 1).

Normally, the toxicity of Cr has some relation with its oxidation states. No toxicokinetic data are available on Cr metal and Cr(II) compounds. An oral-dose-finding study indicated that water-soluble Cr(III) compounds only exhibited moderate toxicity due to the relatively higher LD50 values (40–422 mg/kg) of Cr(III) for rats and mice, whereas Cr(VI) was evaluated as a carcinogenic species [2], and Cr(VI) compounds are thought to be toxic for both plants and animals at low concentrations [3].

The major Cr discharged from the metal industry is Cr(III), and Cr(VI) in industrial wastewaters mainly originates from tanning and painting. As one of the toxic heavy metals which exists stably in nature, Cr(VI) is more toxic than Cr(III). In an aqueous medium, Cr(VI) is presented as CrO_4_^2−^ or Cr_2_O_7_^2−^. Up to now, Cr(VI) species originating from Cr-containing electroplating wastewater have become a typical primary water pollutant. River water was estimated to contain only approximately 1 ppb of Cr, but it was a long time ago. The Cr content has dramatically increased in recent years, for example, 6–12 ppb in the Godavari River in 2014 [4] and 10.5–113 ppb in the Yangtze River in 2009 [5] have been reported. The consistent diffusion of Cr(VI) into groundwater was also detected [6]. Undoubtedly, Cr species would have a great influence on the drinking-water quality and public health.

Due to the different oxidation states of Cr, the selective removal of Cr species from sewage water is therefore difficult to operate. The conventional physical and chemical treatment techniques are usually costly and consume a high amount of energy, they produce harmful by-products, and they can cause secondary pollution. Usually, Cr(III) could be precipitated as hydroxide; however, coagulation is not a very practical method for Cr(VI) removal due to the problems aroused by the disposal of Cr(VI)-containing solid waste. The employment of iron sulphate (FeSO_4_) is also a good choice to reduce Cr(VI) to Cr(III); however, the practical application of this method in drinking-water production seems infeasible.

Currently, the Cr removal from water is necessary. Ion exchange [7,8] and adsorption [9] can be applied for this purpose. As a well-known equilibrium separation process, adsorption has been an effective method for water-decontamination applications and is superior to other technologies. Moreover, adsorption also does not produce any additional harmful substances. The present review article hopes to analyze and discuss the research progress and perspectives of Cr species removal from wastewater by adsorption. The main aim of this review is to provide a summary of the most recent information on the research and development of multifunctional and efficient sorbents. For this purpose, a wide range of lists for the reported adsorbents were compiled. The adsorbents’ properties, maximum adsorption capacities and effects of condition parameters (solution pH, initial adsorbate concentration, adsorbent dosage, contact time and temperature) on the adsorption procedures were critically discussed in detail.

## 2. Cr Removal: The Developed Adsorbents

To overcome problems associated with Cr species pollution, lots of new functional materials have been developed and widely used (Table 1). The practical application of versatile adsorbents has also been carried out, providing water security for people of different regions.

### 2.1. Carbon Materials

Carbon materials, including active carbons (ACs), carbon microsphere (CMS), carbon nanotubes (CNTs), graphene (Gr) and graphene oxide (GO), are of great importance in adsorption fields. In particular, ACs and CMS have been extensively used in various industrial adsorption and separation processes for the treatment of metal ions, such as lead (Pb), copper (Cu), mercury (Hg) and so on, due to their cost-saving and highly efficient performance. Additionally, the excellent stability and the multifunctionality of carbon-material shave guaranteed their wider and practical applications in Cr removal.

#### 2.1.1. ACs

Due to its excellent physicochemical properties and the availability of providing abundant adsorption sites, ACs have been used for the adsorption removal of Cr species. Specially, the massive production of ACs by activation of particular agricultural wastes, such as peanut shells [10], and hard-to-dispose wastes, such as waste rubber tires [11], has been successfully carried out, opening up some new opportunities for AC based adsorbents. Usually, the micropores in ACs could adsorb Cr(VI) via physical interactions such as the Van de Waals force [12,13]. To fulfill the highly efficient removal of Cr species, physical adsorption was not enough. Thereupon, the further modification of ACs was carried out. The introduction of various functional groups onto ACs could provide additional adsorption sites for more efficient adsorption [14,15]. The existence of different adsorption sites at ACs which were suitable for positively charged metal ions or negatively charged Cr(VI) [16]. Normally, positively charged adsorbents usually possess relatively higher adsorption capacity toward Cr(VI). For example, quaternary amine-anchored AC (AC-NH_2_) exhibited a much higher adsorption capacity of 112.36 mg/g for Cr(VI) than that (26.25 mg/g) of as-received AC [17].

The coexistence of Cr(VI) and humic acid (HA) in wastewater is obvious. The concurrent adsorption of Cr(VI) and HA by powdered AC was evaluated, and the adsorption of both HA and Cr(VI) could be enhanced with increasing levels of either HA or Cr(VI) [18]. Recently, more and more biomass-based ACs have been fabricated for Cr species removal, providing new thoughts for developing novel low-toxicity, cost-saving and easy-to-operate bio-adsorbents [19,20,21].

#### 2.1.2. CMS

The small pore sizes and low pore volume of ACs usually limit their wider applications due to the slower mass transport kinetics. Mesoporous carbons, such as CMS, were therefore developed and believed to be a kind of promising adsorption material. Due to its relatively bigger pores, well-developed mesoporous channels and larger pore volumes which are beneficial to accommodating more or larger ions, the diffusion of Cr(VI) in CMS would be greatly accelerated. Due to its larger surface area, the adsorption capacity (Q_m_) of mesoporous carbon microsphere (MPCMS) for Cr(VI) reached 165.3 mg/g [22]. The combination of CMS with aminated sodium lignosulfonate could produce novel layered N-containing a carbon–lignin-based adsorbent, which exhibited 99% Cr(VI) removal under neutral conditions [23]. Magnetic carbon microspheres (MCMSs) showed 100% Cr(VI) removal from a Se(IV)-Cr(VI) coexisting system in 120 min [24].

Novel mesoporous composites of goethite/carbon microspheres (α-FeOOH/CMSs) and akaganeite/carbon microspheres (β-FeOOH/CMSs) were employed to selectively remove Cr(VI); the coexistence of Cl^−^, Cu^2+^ and Ni^2+^ would not cause significant negative effect, whereas SO4^2−^ and PO_4_^3−^ would hinder the effective removal [25]. CMS prepared from powdered sodium lignosulfonate and polystyrene could be used for Cr(VI) removal without adding any binder, and its high adsorption capacity toward Cr(VI) (227.7 mg/g at an initial pH value of 2) made it very attractive [26].

#### 2.1.3. CNTs-Based Composites

Owing to its merit of having a very high specific surface area, CNTs have displayed a high adsorption capacity and fast adsorption rate toward various organic contaminants (phenols, dyes, antibiotics, and so on) and metal ions (Pb, Cu, Cr, etc.). Interestingly, the CNTs systems after adsorption of Cr(VI) exhibited less toxicity and carcinogenic activity [27]. Hydroxylated and carboxylated CNTs possess different surface functional groups, such as hydroxyl or carboxyl groups, which exhibited a higher Cr removal efficiency than pristine CNTs, confirming that the modification of CNTs is beneficial [28,29]. To quickly remove Cr(VI), polyamine-modified carbon nanotubes (PA-CNTs) were developed, and a high Cr(VI) adsorption capacity of 168.54 mg/g, as well as ultrafast removal, could be detected due to the introduced amine groups on the surface. Interestingly, its adsorption capacity increased to 392.78 mg/g in the presence of Rhodamine B [30]. To facilitate the recovery of CNT-based composites, a high-ferromagnetism Fe_3_O_4_/CNT composite possessing adsorption capacities of 47.98~83.54 mg/g for Cr(VI) and magnetic separation ability was developed [31].

The utilization of CNTs-based adsorbents for future practical applications should be evaluated. Therefore, the separation behaviors of a chitosan–multiple-walled CNTs–iron (CS–MWCNTs–Fe)-based continuous-flow column was tested, and around 54% Cr(VI) removal could be achieved (water flow rate = 1 mL/min, feed Cr-VI concentration = 30 mg/L, packed bed height = 8 cm) [32]. The vertical and uniform growing of CNTs on carbon nanofibers (CNFs) could enhance the specific surface area (398.127 m^2^/g) and create a higher adsorption capacity toward Cr(VI) (150 mg/g) within an hour, and 227 mg/g at equilibrium could be observed [33]. However, the recyclability of CNTs–CNFs needs to be improved due to the fact that the vertical form of CNTs would be easily damaged. MWCNTs modified by iron–manganese binary oxide (FeMnO_x_/MWCNTs) showed superior adsorbability toward Cr(VI) in a binary system of Cr(VI) and As(III) due to the electrostatic attraction, the redox and ligand exchange [34]. The regeneration studies showed that FeMnO_x_/MWCNTs had good reproducibility and a good recycling performance, which is also critical for the possible practical application. The further introduction of mono-tosyl-β-cyclodextrin (MT-β-CDTs) onto MWCNTs/Fe_3_O_4_ would increase its adsorption capacity toward Cr(III) [35]. A natural organic polymer, chitin-modified MWCNTs/Fe_3_O_4_, was also developed, which possessed a removal efficiency of 99.1% toward Cr(VI) [36], again indicating that the introduction of organic components onto CNTs was beneficial to their adsorbability toward Cr(VI). In addition, the modification of CNTs with special molecules would enhance the adsorption selectivity. For example, the sensitive and highly selective removal of 90% of Cr(VI) by magnetic CNTs functionalized with imidazolium ionic liquid could be carried out, even at a level of ppb and in the presence of high concentrations of other cations, such as Hg^2+^ and Cd^2+^, or anions such as NO^3−^ and SO_4_^2−^ [37].

#### 2.1.4. Gr and GO-Based Composites

Significantly great attraction was shown for the design and development of efficient and robust adsorbents with outstanding stability, namely Gr and GO-based versatile adsorbents, for the removal of environmental pollutants. Pristine Gr and GO themselves could be used as highly efficient adsorbents; for example, GO exhibited a high adsorption capacity (366.3 mg/g) toward Cr(III) [38]. However, it is very difficult to separate/recover Gr and GO after adsorption, leading to secondary pollution. Fortunately, Gr/GO compounded with other materials could output versatile nanocomposites, which might be used for the selective adsorption of different contaminants (metal ions, organic dyes, pesticides, medicines, etc.) due to the introduced various surface functionalities. In addition, these nanocomposites are usually more easily recovered, which might show more promising practical applications in wastewater treatment [39]. The performance of GO/Gr-based composites depends on the active sites and functionalities on the surface [40]. To exploit the maximal efficacy of these composites, different components have been introduced onto GO/Gr via multifarious approaches, such as covalent coupling, self-assembly, electroless plating and so on.

**Figure 2:**
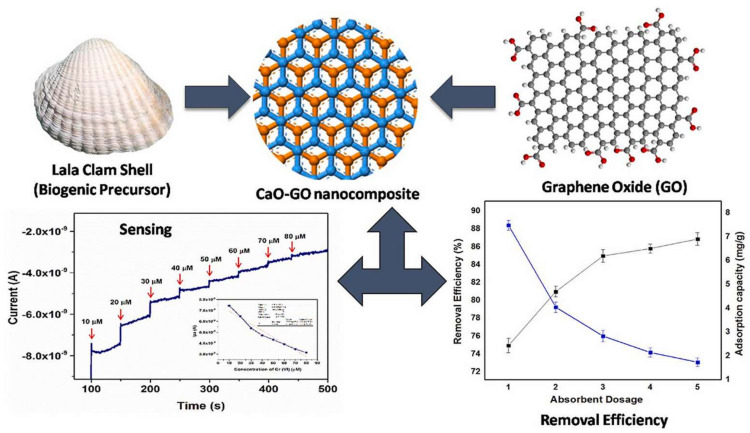
CaO NPs cross linked GO for Cr(VI) removal and sensing [41]. Copyright belongs to Elsevier B.V.

Metal-based adsorbents possess lower adsorption capacities and slower adsorption kinetics for Cr(VI). However, the combination of a metallic compound with GO/Gr could effectively overcome these deficiencies, as well as improve the recovery properties of Gr/GO-based composites. The immobilization of CaO nanoparticles (CaO NPs) from Lala clamshells onto GO was carried out, and the obtained biogenic CaO-GO exhibited an adsorption capacity (38.04 mg/g) for Cr(VI) removal (Figure 2). Obviously, it could be used as a sensitive sensor for Cr(VI) sensing besides high adsorption capacity (38.04 mg/g) and removal efficiency (~90%) for Cr(VI). A combination of external mass transfer and chemisorption might devote to the adsorption [41]. When zinc oxide (ZnO) NPs were decorated onto GO, the mobility of Cr(VI) in the composite was improved, facilitating Cr(VI) adsorption kinetics and enhancing removal efficiency [42]. The simultaneous combination of Ca and Zn with GO was also conducted, and the GO@CZ nanocomposite demonstrated high adsorption capacity of 285.71 mg/g for Cr(III) at pH = 7 [43]. It is worth noting that when organic components were incorporated into GO-metal-based materials, the removal efficiency and adsorption capacity would be significantly improved [44,45]. Poly(dimethyl diallyl ammonium chloride) (PDMDAAC)-modified magnetic GO (GO/Fe_3_O_4_) exhibited a high adsorption capacity (95.2 mg/g) toward Cr(VI) due to the electrostatic attraction by quaternary amine [46]. Polypyrrole (PPy)-anchored Gr-silica nanosheets (Gr-Si-PPy) possessed a high specific surface area, and they exhibited a much higher adsorption capacity for Cr(VI), i.e., 429.2 mg/g at 298 K (pH = 2) [47].

The assembly of GO with other nanosheets, such as MoS_2_, would produce novel composites with a higher specific surface area and higher removal efficiency. MoS_2_/rGO with an adsorption capacity of 80.8 mg/g toward Cr(VI) was therefore fabricated [48]. Although a relatively lower maximum adsorption capacity of 43.95 mg/g for Cr(VI) at a rod-like nano-MoS_2_/GO nanocomposite could be obtained, the adsorption selectivity toward Cr(VI) in the coexistence of organic contaminants was significantly enhanced [49]. To improve the surface area of metal and Gr/GO-based adsorbents, novel sandwich-like GO@SiO_2_@C@Ni composites were developed, and GO@SiO_2_@C@Ni-400 sintered at a carbonization temperature of 400 °C exhibited excellent dispersibility, a large surface area (229.88 m^2^/g) and high saturation magnetization, revealing a high adsorption capacity (299.20 mg/g) toward Cr(VI) [50]. If GO- and NiFe-layered double hydroxide were hydrothermally treated, a novel three-dimensional (3D) hierarchical GO-NiFe LDH composite with a sandwich-like, highly porous and well-ordered structure would be fabricated, and it would be more stable and easier to recycle [51]. The hybridization of nZVI with LDH decorated rGO, ternary (Fe@LDH/rGO) composite with better dispersibility, improved hydrophilicity and more positive surfaces would be produced, which possessed higher removal efficiency and capacity for Cr(VI) oxyanions [52], which was superior to nZVI-rGO [53], indicating the introduction of LDH was beneficial. The idea and practical operation of 3D composites continued to evolve in recent years, which offered a lot of possibilities for design and fabrication of novel useful adsorbents [54].

Fe_3_O_4_/GO nanocomposite was also employed for the effective adsorption of Cr(VI) from aqueous solution, and the magnetic separation of nanocomposite from solutions after adsorption was easily realized [55]. When CS was introduced onto Fe_3_O_4_/GO nanocomposite, its adsorption performance would be greatly changed, indicating the combination of organic components with GO or GO-based composites might be a very exciting research area [56]. More and more researchers have focused on the development of novel multifunctional GO/Gr-based adsorbents by introducing various organic components. GO/CS composite was crosslinked with 3-glycidoxypropyltrimethoxysilane (KH-560) to form a three-dimensional (3D) GO/CS/KH-560 aerogel with porous network structure, which possessed an equilibrium adsorption capacity of 146 mg/g toward Cr(VI) in within 120 min [57]. The removal of Cr(VI) in the presence of Hg(II) from aqueous solutions by 4-amino-3-hydrazino-5-mercapto-1,2,4-triazole covalently bonded GO was evaluated; the tremendous adsorption capacities toward Cr(VI) (734.2 mg/g) and Hg(II) (1091.1 mg/g) were even higher than pure GO, indicating the introduced multidentate ligands was really conducive [58]. Similar research was carried out by grafting poly(allylamine hydrochloride) (PAH) onto amino-modified GO, and the resulting PAH-ASGO also exhibited high adsorption capacity of 373.1 mg/g for Cr(VI) [59]. PAH-ASGO could especially effectively reduce Cr(VI) from a high concentration level (9.9 mg/L) to an extremely low level of 0.004 mg/L in a very short time of 10 s, and its excellent reusability was also significant [59].

**Figure 3:**
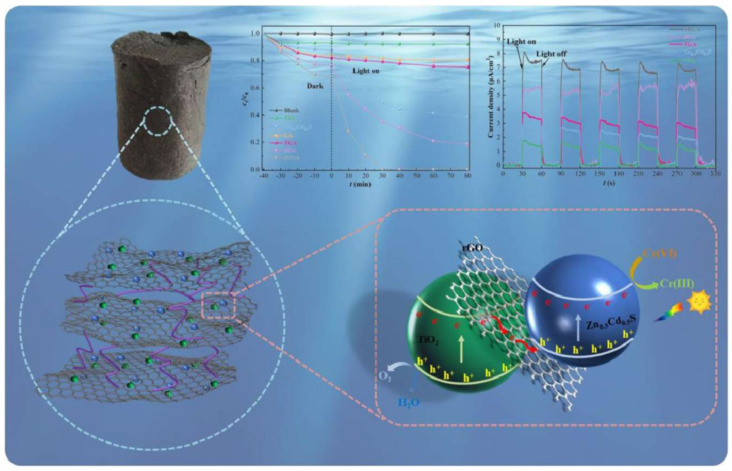
Efficient removal of Cr(VI) by TSGA via the synergy of adsorption and photocatalysis [60]. Copyright belongs to Elsevier B.V.

The electrons’ transfer ability of different composites based on rGO, GO and Gr sheets could be promoted; therefore, their photocatalytic ability would be greatly enhanced, which could be employed to increase the Cr-removal effectiveness through the synergistic effect. By employing reduced graphene oxide (rGO) as a charge-transfer mediator, a three dimensional (3D) photocatalyst [TiO_2_-Zn_x_Cd_1−x_S rGO aerogel (TSGA)] fabricated by combining TiO_2_ and Zn_x_Cd_1−x_S with Gr aerogel could aqueously remove Cr(VI) (*R* = 100%) via adsorption, along with visible light (wavelength > 420 nm) photocatalysis in 30 min (Figure 3) [60].

### 2.2. Silicon-Based Materials

Various silicon-based porous materials, including crystalline metallosilicates (zeolites and zeotype materials), silica (microporous, mesoporous and microporous), silica gels and so on, have been of tremendous importance for the adsorption methodology besides ACs and CMS. However, the large-sized silicon-based adsorbents always exhibit a relatively lower adsorption capacity, which greatly limits their possible wider applications. The nano-crystallization of materials is helpful to improve their specific surface area, thus improving the adsorption efficiencies. Compared with as-prepared mesoporous silica–carbon (MSC), nanoscale zero-valent iron (nZVI)-particles-embedded MSC (nZVI-MSC) exhibited a more ordered structure, larger surface area and much higher Cr(VI) removal efficiency [61]. The treatment of attapulgite with HCl or HF of different concentrations could regulate the morphology and composition [62]. Further characterization of silicon-rich biochar-supported nanoscale zero-valent iron (nZVI) was performed, and ferrous chromite (FeCr_2_O_4_) on the surface could be detected, indicating that the reduction and adsorption both contributed to the removal of Cr(VI) [63]. By immobilizing the nZVI composite onto modified attapulgite, the enhanced Si-O-Fe coupling mediated by silicon would occur, resulting in the reduction of Cr(VI) and higher Cr(VI) removal efficiency. The nanosilver-assisted chemical etching (Ag-ACE) of kerf loss silicon waste was performed, which was functionalized with 3-aminopropylethoxysilane (APTES) to obtain a hybrid material, APTES-NPSi, for the highly effective removal of Cr(VI) [64]. Interestingly, its adsorption capability was highly pH dependent.

The introduction of specific functional groups onto silicon-based materials was also conductive to regulate their adsorption performances. For example, the introducing alkylamine chains onto porous silica beads could enhance its adsorption capacity toward Cr(VI) due to the abundant interfacial amino groups [65]. Thereafter, calcium silicate hydrate prepared by coal gangue was used to adsorb Cr(VI), and a relatively higher adsorption capacity, i.e., 68.03 mg/g, could be obtained [66].

### 2.3. Resins

Resins are not only used as adsorbents for the removal of contaminants, but they are also widely applied to recover valuable substances, such as Cr(VI), from electroplating wastewater. To eliminate toxic Cr species, the rational selection of resins with specific functional groups (for example, C=S, C-S, C-N, -NH_2_, -CHO, -OH, etc.) and suitable pores is very important [67]. For example, the resins with hydrophobic properties exhibited low adsorption selectivity, which could be improved by surface modifications [68,69].

By combining ion-exchange and precipitation-reduction mechanisms, a strong-base silica-supported pyridine resin (SiPyR-N4) was developed to remove/recover Cr(VI) from electroplating wastewater. Rapid Cr(VI) removal (99.3% removal within 5 min from a 100 mg/L Cr(VI) solution) could be realized, demonstrating that the ion exchange by SiPyR-N4 was really efficient [70]. By in situ polymerization of different monomers, including epichlorohydrin (ECH), dimethylamine (DMA) and ethylenediamine (EDA), with the weakly alkaline anion-exchange resin D301, the modified anion-exchange resin (EDE-D301) possessed enhanced surface area, positive charge and high contents of N and Cl, facilitating the highly efficient adsorption of Cr(VI) (capacity of 298 mg/g, and 93% removal after four consecutive adsorption–desorption cycles) [71]. Similarly, a styrene-based ion-exchange D201 resin-hydrated iron oxide (HFO) nanocomposites for Cr(VI) removal was investigated, and the efficient removal of Cr(VI) from 10 mg/L to below 50 ppb could be carried out [72]. Macroporous strong acidic cation-exchange resin (D001) modified by nanosized goethite (nFeOOH@D001) also exhibited much better ability (80.2% removal and capacity of 7.4 mg/g) to remove Cr(VI) than D001 [73]. Further modification of sulfide onto nZVI supported porous D201 could improve its removal performance (89.67% removal and capacity of 137.35 mg/g for 100 mg/L of Cr(VI) solution), recycling performance and longevity [74]. The possible adsorption mechanisms of nZVI-modified resins, including Amberlyst 15 or resin D201, were investigated, and the simultaneous reduction of Cr(VI) to Cr(III) might be implemented for the efficient cation-exchange-based Cr removal [75,76]. The evaluation of quaternary amine functional groups of anion-exchange resins for Cr(VI) removal was further implemented, and different ion-exchange capacities of 280.25 mg/g, 147.67 mg/g and 163.67 mg/g for Eichrom 1-X4, Lewatit M+ M800 and Lewatit A8071 could be observed, respectively [77].

The removal of trace Cr species is really a huge challenge. To overcome the shortages of conversional resins which only possess high removal efficiencies for Cr species of high concentrations, a weak base anion-exchange resin, Duolite A7, was developed for trace Cr(VI) removal. Extensive investigations revealed that redox reactions occurred besides ion exchange, and the produced Cr(III) could be precipitated inside the resin or bound to the carboxylic groups outside due to the high-affinity interaction [78].

### 2.4. Iron-Based Adsorbents

Recently, the reaction of Cr(VI) with ferric and ferrous compounds has received as much attention due to the assumption of strong interactions between Cr(VI) and the oxide surface [79]. Because of the slow redox transformation of Cr(VI), it is important to understand the reactions of Cr(VI) and iron oxides in various systems. For example, ferrihydrite (Fh) was used as an adsorbent for Cr(VI) removal from organic ligands and Cr(VI)-containing wastewater, and the removal efficiency could be improved by adding aluminum (Al) to increase its specific surface area, indicating that the surface complexation and electrostatic attraction played an important role in the adsorption [80]. Moreover, sulfonated nanoscale zero-valent iron (S-nZVI), together with organic acids, including citric acid and oxalic acid, exhibited a significant promotion of Cr(VI) removal due to the synergistic effects of adsorption and reduction [81]. Other iron-based materials, such as ferrous sulfide (FeS), could be used to effectively remediate Cr pollution [82]. To improve the adsorption capacity of iron-based adsorbents, many tentative works have been performed in the past few years.

#### 2.4.1. Iron Oxyhydroxide (FeOOH) and Its Derivatives

Owing to the high specific surface area and abundant surface hydroxyl groups, iron oxyhydroxide (FeOOH)-polymorphs-based nanoparticles, including goethite (α-FeOOH), akaganeite (β-FeOOH), lepidocrocite (γ-FeOOH) and feroxyhyte (δ-FeOOH), showed strong electrostatic interactions toward Cr(VI) [83]. The δ-FeOOH might be used more widely in the treatment of wastewater due to its ferromagnetic properties and ability to easily separate/regenerate [84]. A ferrate K_2_FeO_4_ could be decomposed to γ-FeOOH/γ-Fe_2_O_3_ in the presence of Mn(II), and the reduction of Cr(VI) could be carried out, facilitating the more efficient removal (97.7%, *C*_0_ = 10.0 mg/L) of Cr(VI) [85].

Nano iron oxides (Fe_2_O_3_) and ferrites have also been intensively used for wastewater treatment [86]. The newly developed very thin and porous α-Fe_2_O_3_ nanofibers exhibited a maximum adsorption capacity of 16.17 mg/g for Cr(VI) removal, and it can be practically utilized due to its highly efficient and low-cost properties [87]. The cost-saving adsorbents are always expected, and industrial wastes, such as foundry sands from the iron foundry industry, could be used for the removal of Cr (VI) [88], providing some references for developing cheaper sorbents.

#### 2.4.2. Magnetic Adsorbents

It is difficult to regenerate traditional adsorbents due to their sinking properties or excellent dispersibility, inevitably causing secondary pollution. To overcome these shortcomings, magnetic materials were developed as novel recyclable adsorbents. The adsorption capacities of magnetite nano-Fe_3_O_4_ particles toward Cr(VI) are usually very low (usually below 5 mg/g) due to the fact that the uptake was mainly composed of physicochemical processes, which greatly limits their widely practical uses [89]. The adsorption capacity of an adsorbent would increase as its specific surface area (SSA) increases [90]. The monodispersed Fe_3_O_4_ hollow microspheres showed a high adsorption capacity of 180 mg/g toward Cr(VI) [91]. The increase in the adsorption capacity could also be expected through the modification or introduction of various new components onto nano-Fe_3_O_4_. For example, the NH_2_-functionalized nano-Fe_3_O_4_ composites exhibited a maximum sorption capacity of 25.58 mg/g for Cr(VI) [92], and the ethylenediamine (EDA)-functionalized magnetic composite showed an improved capacity of 32.15 toward Cr(VI) [93].

Furthermore, the increase in surface area is beneficial to the adsorption and reduction ability of magnetic nano-adsorbents. As-prepared MnFe_2_O_4_ microspheres with myrica rubra-like structures exhibited an excellent catalytic reduction ability for Cr(VI) removal from wastewater that was much better than commercial MnFe_2_O_4_ due to its smaller sizes (~17 nm) and highly promoted catalytic reduction performance [94].

The basic adsorption principles for Cr(VI) expand from ion exchanges to electrostatic gravitations and coordination interactions [95]. Based on these findings, diethylenetriamine (DETA), triethylentetramine (TETA) and tetraethylenepentamine (TEPA) were also used to functionalize nanomagnetic adsorbents, and their adsorption capacities were increased to impressive levels of 149.25, 204.08 and 370.37 mg/g, respectively [96]. The polymerization of glycidylmethacrylate (GMA) onto Fe_3_O_4_ nanoparticles would produce magnetic polymer microspheres (MPMs), which were further modified with EDA (EDA-MPMs) to reach a high maximum adsorption capacity of 253.2 mg/g for Cr(VI), indicating that the introduced multiple functional groups were useful [97].

The combination of magnetic particles with various sorbents would slightly decrease their adsorption capacities due to the low adsorption capacity of the inorganic magnetic particles. However, a good regeneration ability would be achieved as a compensation. For example, Fe_3_O_4_-loaded MCMs could be rapidly separated and regenerated in contrast with pristine MCMs, and a preferable capacity (123.9 mg/g) would be maintained even after five adsorption–desorption cycles compared with its initial capacity of 156.3 mg/g [22]. Polypyrrole (PPy)-coated secondary fly-ash–iron (PPy-MSFA) composites were fabricated; they were floatable and exhibited adsorption capacities of 119.33 mg/g for Cr(VI) [98]. To more conveniently recover the adsorbents, novel magnetic carbon microspheres, which are carbon nanofibers (CNFs) decorated with iron nanoparticles (nanofurry microspheres), were developed, and they exhibited easy magnetic separation capability [99].

To improve its biocompatibility, versatile biodegradable components could be incorporated with a sorbent. Meanwhile, the multifunctional groups of any biodegradable component are beneficial to improving its adsorption capacity. Biomaterials such as corn straw [100], CS [101,102] and so on were grafted onto nano-Fe_3_O_4_, and the obtained magnetic biocomposites exhibited both higher adsorption capacities and better biocompatibility. In addition, the adsorption capacity of magnetic CS composites (Fe_3_O_4_@CS) toward Cr(VI) gradually increased with an increase in loading proportion of CS, indicating that the organic component of the magnetic sorbent dominated its adsorption effectiveness [103].

### 2.5. Polymers

Various adsorbents have been extensively investigated and utilized for Cr(VI) removal. Due to the lack of specific Cr-adsorptive functional groups or heaviness, their adsorption capacities are relatively lower, which greatly limits their practical applications. To obtain new adsorbents with larger Cr(VI) adsorption capacities, versatile polymers or macromolecules have been further developed, and the Cr removal efficiency has been improved. For example, the cationic group contents in polyacrylamide (PAM) was found to benefit the adsorption of Cr(III) [104]. A copolymer hydrogel prepared by 2-hydroxyethyl acrylate (HEA) and 2-acrylamido-2-methylpropane sulfonic acid (AMPS) exhibited a priority adsorption order of Cr(III) > Fe(III) > Cu(II) > Cd(II) > Pb(II) in a multicomponent solution [105].

The combination of polymers with other base materials, such as the introduction of inorganic components into polymers, is beneficial. Highly porous hydrophilic polyacrylonitrile (PAN)-membrane-supported nano zero-valent iron (NZVI) was fabricated, and the further graft of glutathione (GSH) could promote its adsorption capacity for Cr(VI); its removal efficiency could maintain over 86% after six adsorption–desorption cycles [106]. To relieve the serious agglomeration of FeS, carboxymethyl cellulose stabilized FeS@extracellular polymeric substance (CMC-FeS@EPS) was developed [82]. The high Cr(VI) removal efficiency (98.00%) in a wide pH range (4–9), as well as the strong anti-interference in the coexistence of ions, guaranteed its potential practical applications. In addition, sulfidated nanoscale zero-valent iron@EPS (SnZVI@EPS) also exhibited a higher adsorption capacity toward Cr(VI) (64.90 mg/g) in a wide pH range, under various conditions (anaerobic or aerobic), and with the coexistence of a high concentration of other ions [107]. Polyethyleneimine (PEI)-functionalized magnetic hydrochar adsorbent exhibits an excellent adsorption capacity of 287.7 mg/g for Cr(VI). L-cysteine-doped polypyrrole-modified bentonite (L-cys/PPy/BT) showed higher adsorption capacities for the removal of Cr(VI) (318.5 mg/g) [108], whereas crosslinked PEI exhibited a high removal efficiency (89~99%) and excellent selectivity and reusability toward Cr(III), even if it was applied to real wastewater samples [109], indicating that the chemisorption usually dominated the adsorption processes. To enhance the adsorption selectivity, the combination of metal–organic framework (MOF) with polyvinylpyrrolidone (PVP) was implemented, and a more significant adsorption effectiveness, as well as higher selectivity, could be obtained due to the improved hydrophilicity and porosity [110]. Through a surface-initiated atom-transfer radical polymerization process, silica-di-block polymer hybrids were prepared by using a hydrophobic monomer (butyl methacrylate, BA) and a hydrophilic monomer (2-(dimethylamino)ethylmethacrylate, DMAEMA) [111]. The hybrids with larger DMAEMA-to-BA mass ratios possessed higher adsorption capacities toward Cr(VI), indicating that N-containing groups contributed a lot to the efficient adsorption. Recyclable calcined CoFe-LDH/g-C_3_N_4_ [112], 3D hierarchical GO-NiFe LDH, hierarchical porous Ni/Co-LDH hollow dodecahedron [113] and so on have been developed based on structural synergistic effects and interpenetrating networks, facilitating their future potential utilization in practical applications.

Microplastics have been recognized as one of the pollutants, but aged microplastics could also be used for the removal of heavy metals to achieve waste utilization and recyclable development. To evaluate the adsorption properties of aged microplastics toward heavy metals, the adsorption of Cu(II) and Cr(VI) by aged polystyrene (PS) and polyvinyl chloride (PVC) was investigated, providing some references for future trends in sustainable development [114]. Green strategies have been increasingly used in developing novel adsorbents. A sodium polyacrylate hydrogel (PAAS) loaded with Fe(III) could generate Fe(II) to reduce Cr(VI) by 93.90–98.25%, which is efficient and eco-friendly for Cr(VI) removal [115].

### 2.6. Layered Double Hydroxides (LDHs)

The high adsorption capacity of an adsorbent is also one of the most important issues for water treatment. A class of two-dimensional materials containing positively charged layers (Al^3+^, Mg^2+^, Zn^2+^, etc.) and counter-anions (Cl^−^, NO_3_^−^, CO_3_^2−^, and SO_4_^2−^, etc.), layered double hydroxides (LDHs), has been extensively utilized as useful adsorbents due to large specific surface area, unique layer structure with abundant bonding sites. For example, the adsorption capacity of Zn/Al-LDHs toward Cr(VI) was over 120 mg/g [116]. Taking advantage of laboratory wastewater containing Cr(III), a novel Mg/Al/Cr layered compound (Mg/Al/Cr(III)-LDH) was developed for the removal of Cr(VI) from laboratory wastewater, and it had an adsorption capacity of 237.80 mg/g [117]. By in situ grown polyaniline (PANI) on Mg/Al LDHs, the organic–inorganic hybrid material exhibited an outstanding removal capacity toward Cr(VI) (393.70 mg/g) from aqueous solution in contrast with that (198.67 mg/g) of PANI nanotubes, providing an ideal material for the treatment of Cr(VI) wastewater [118]. From the point of view of a classic ion-exchange reaction, a single-phase material of Mg_0.66_Al_0.34_(OH)_2_(Mo_3_S_13_)_0.03_(NO_3_)_0.14_(CO_3_)_0.07_∙H_2_O (Mo_3_S_13_-LDH) was rationally designed by intercalating Mo_3_S_13_^2−^ into the MgAl-LDH gallery for specifical trapping Cr(VI) in the coexistence of multiple interfering anions [119]. The excellent structural stability of Mo_3_S_13_-LDH guaranteed its possible practical applications in the future.

To realize the synergetic effect of adsorption and photocatalysis, a novel CuBi_2_O_4_/calcined ZnAlBi LDHs (CBO/CLDHs) composite was fabricated for Cr(VI) removal, and a removal efficiency of above 95% toward Cr(VI) (40 mg/L) could be observed [120]. Meanwhile, Cr(VI) was gradually reduced to Cr(III) under visible-light irradiation. An iron–manganese LDH (MnFe-LDH)- and MnFe_2_O_3_-anchored three-dimensional porous carbon nanofiber (MnFe-LDH/MnFe_2_O_3_@3DNF) could effectively reduce high levels of Cr(VI) to meet the guideline concentrations for potable water supplies [121]. The newly developed nanostructures, inner transition metal ions doped LDHs embedded CNTs, could selectively adsorb Cr(VI) at ultra-low trace levels due to the improved synergistic catalytic properties, which could be employed for highly efficient sensing Cr(VI) [122]. A robust sorbent, Co-Al-LDH@CS/Fe_3_O_4_, was fabricated by the combination of Co-Al-LDH with magnetically interspersed CS and cystamine, and its higher adsorption capacity toward Cr(VI) (710.79 mg/g) confirmed the existence of positive synergistic effects and abundant affinity sites (-SH and -NH_2_) on the surface [123].

Recently, an effective approach based on defect engineering was developed for the fabrication of lignin-derived carbon and Ca/Fe/Al-trimetallic LDH (LDH@LDC) composite [124]. Due to the defects on the LDH layer from the H_2_ plasma treatment, LDH@LDC exhibited outstanding removal efficiency for Cr(VI) and U(VI) by enhanced ion exchange [124].

### 2.7. Titanates

Due to the high ion-exchange capacities and excellent stability, titanate-based materials have been utilized as efficient adsorbents to remove various contaminants from wastewater. Similarly, a better adsorption performance would be obtained when other multifunctional organic components are introduced onto titanates [125]. A core–shell structure made from polyaniline/hydrogen-titanate nanobelt (PANI/H-TNB) composite showed an excellent adsorption capacity of 156.94 mg/g toward Cr(VI) under various environmental conditions [126]. Additionally, the satisfactory reusability of PANI/H-TNB composite would greatly reduce the expenses of wastewater treatment. CS-based hydrogel was further incorporated with titanate- and carbon-dots-modified cellulose nanofibers, which could effectively remove Cr(VI) with higher adsorption capacity of 228.2 mg/g [127]. Definitely, the fabrication procedure and the composite were relatively too complicated.

The complete Cr(VI) removal from contaminated water could be implemented by simultaneous reduction of Cr(VI) and immobilization of Cr(III), using sulfonated carbon hemisphere (CHS)-supported mesoporous TiO_2_ nanoparticles, and the exceptional reduction efficiency, as well as the ion exchange by -SO_3_H^+^ on CHS, contributed to the removal of reduction of Cr(VI) and the sequestration of Cr(III), respectively [128]. The binary composites of PEI (an interior core)-anchored titanate nanotubes (TNTs, an exterior shell) also exhibited high Cr(VI) adsorption capabilities due to the electrostatic attraction, redox reaction and chelation [129].

The combination of inorganic components onto titanates is also beneficial. Abundant oxygen vacancies could be formed on the surface of lanthanum-doped bismuth titanate nanosheets; thus, the activated nanosheets exhibited a stronger photocatalytic ability, and the efficient synchronous removal of Cr(VI) and methyl orange could be successfully performed [130]. The encapsulation and homogenous dispersion of FeS NPs into the interlayers of titanate nanotubes were carried out, and highly efficient removal of total Cr was obtained [131]. The reduction of Cr(VI) to Cr(III) by FeS NPs and the adsorption of Cr(III) by titanate nanotubes both contributed a lot to the Cr(VI) removal.

### 2.8. Reductive Adsorbents

To reduce heavy metal ions to low levels, reductive materials have shown some advantages, such as high adsorption effectiveness, the use of inexpensive adsorbent materials, reducing the toxicity of metal ions and so on [132]. Lignite, brown coal and kerogen could reduce Cr(VI) to Cr(III), and various oxygen-containing functional groups, such as C=O and C-O, would be introduced onto lignite by the Cr(VI) oxidization; hence, Cr species could be removed from aqueous solutions due to the emerging intermolecular forces between Cr(III) ions and the O atoms of C=O and C-O groups in the oxidized lignite [132,133].

Additionally, an adsorption–reduction synergistic effect could be achieved by some adsorbents [79,134]. A ternary magnetic composite containing graphene oxide (GO), diethylenetriamine and Fe_3_O_4_ nanoparticles was developed, and the maximum adsorption capacity of 123.4 mg/g toward Cr(VI) was much higher than that of magnetic GO. The reduction of Cr(VI) to Cr(III) during the adsorption was confirmed by X-ray photoelectron spectroscopy (XPS) analysis, indicating that the Cr(VI) adsorption and the reduction both contributed to the Cr(VI) removal [135].

The Cr(VI)-removal performance could be promoted by combining the excellent adsorption effects and the reduction properties. Fe-ethylene glycol complex microspheres [136], mesoporous amino-group-functionalized iron/silica hollow spheres (Fe/SiO_2_-NH_2_ HSs) [137] and 3D porous CoFe_2_O_4_@SiO_2_-NH_2_ [138] have been applied in Cr(VI)-contaminated-water remediation. To realize the resourceful utilization of industrial waste, the Fe-P slag was employed as a reductive agent for Cr(VI) removal by a simplified industrial procedure, providing some new and feasible thoughts for the resourceful utilization of industrial waste in Cr wastewater treatment [139]. Industrial lignin (pulping black liquor lignin (BLN), enzymolytic lignin (ELN) and SPORL pretreatment spent liquor (FS)) showed a maximum adsorption amount of 864.30, 801.57 and 642.26 mg/g toward Cr(VI), respectively, where Cr(VI) was also reduced to low-toxicity Cr(III) [140].

**Figure 4:**
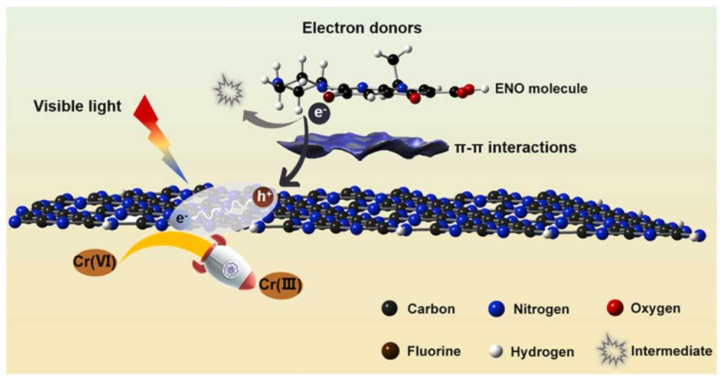
The synergistic removal of pollutants (antibiotics and Cr (Ⅵ)) by g−C_3_N_4_ [141]. Copyright belongs to Elsevier B.V.

Ultra-thin g-C_3_N_4_ itself especially possesses the ability of adsorption and photodegradation because it can act as electron donors (hole sacrificial agents) to accelerate the separation of photo-generated electron–hole pairs and allow more electrons/holes to participate in the reduction of Cr(VI) [141]. The reasonable design of multifunctional materials to enhance the synergistic efficiency proved to be feasible (Figure 4). Furthermore, TiO_2_/g-C_3_N_4_ heterojunctions were developed by evenly grounding and subsequently heat-treating acid-treated H_2_Ti_3_O_7_ nanobelts and superior thin g-C_3_N_4_ nanosheets, and the in situ growth of rhombic TiO_2_ on g-C_3_N_4_ nanosheets effectively decreased the bandgap by adjusting the surface and electronic structures [142]. Notably, a more efficient photoreduction of Cr(VI) could be observed, which was 22 times that of pristine g-C_3_N_4_ nanosheets.

### 2.9. Montmorillonite

Montmorillonite, a clay, has been widely used to remove heavy-metal contaminations by cation exchange in the interlayers and formation of inner-sphere complexes through Si–O− and Al–O− groups. To enhance its adsorption capability, the combination of montmorillonite with other components containing abundant, accessible sorption is essential. For example, CS/montmorillonite nanocomposites exhibited a higher adsorption efficiency, i.e., 45% removal, toward Cr(III) [143]. Various Al-13 cations, dodecyl trimethyl ammonium chloride (DTAC) or dodecyl amine (DA) were used to modify montmorillonite nanoparticles; the as-prepared nanocomposites possessed specific surface areas and high adsorption capacities for Cr(VI) due to the large basal spacing and good porous structure [144].

Taking advantage of the agricultural wastes, a calcined composite from dolomite, montmorillonite and corn stover was fabricated for the aqueous removal of Cr(VI), with removal efficiencies of 84–86% [145].

### 2.10. Biological Adsorbents

The aqueous removal of Cr(VI) by biosorbents has shown some advantageous over the existing conventional physicochemical techniques, therefore it has attracted great attraction in recent years [146,147]. The removal of Cr species from the industrial effluents of electroplating units by the biomass of cyanobacterium was investigated, which showed a rapid rate for Cr adsorption within the first 15 min [148]. Resting cells of fusarium solani [149], traditional Chinese medicine residual (TCMR) [150], chromate-resistant bacterial strain [151], Yarrowia lipolytica cells [152] and living cells of chryseomonas luteola TEM 05 [153], Canadian peat and coconut fiber, Serratia proteamaculans [154], mussel-shell ash or a sludge/ashes waste mixture [155], activated dry flowers (ADF) of Alstonia Scholaris [156], Araucaria leaves [157], rice husk [158], thermal decomposition of wheat straw (BCS) and wicker (BCW) [159], UV-mutant *Bacillus subtilis* [160] and sludge-derived biochar (SDBC) [161] also showed a high adsorption capacity toward Cr(VI). The calcium hydroxide modified mangosteen peel especially exhibited highly selective adsorption for Cr(VI) over other metal ions, such as Pb(II), Fe(III), Zn(II), Cd(II) and Cr(III), and the cost-effective and ecofriendly functionalization process made it useful as a green adsorbent [162]. To remove Cr(VI) from groundwater, Fe-modified BC (FeBC) was used as permeable reactive barriers (PRBs) [163]. The long-period (563 days) tests confirmed its excellent performance in terms of Cr(VI) removal with a relatively low cost. To reduce the costs and increase the efficiency, cheaper biomasses will be utilized, or pyrolysis compounding of different biomasses should be carried out.

**Figure 5:**
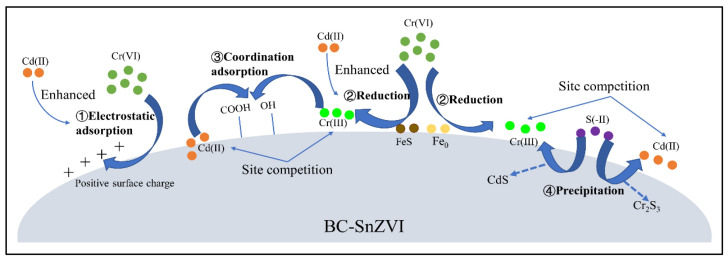
The co-removal of Cd(II) and Cr(VI) by BC-SnZVI [165]. Copyright belongs to MDPI (Basel, Switzerland).

Heteroatom-containing compounds can form coordination bonds with Cr species, so heteroatom (nitrogen (N), sulfur (S), phosphorus (P), etc.)-modified materials would be efficient adsorbents [164]. Sulfide-modified nanoscale zero-valent iron (SnZVI) was further anchored onto BC (BC-SnZVI), which showed an outstanding performance for Cr(VI) removal from a Cr(VI)-Cd(II) binary system (Figure 5) [165]. The amine and hydroxyl groups of CS are predominantly responsible for binding interactions, and chemisorption was therefore involved in the removal of Cr(VI) by Fe_3_O_4_@CS [103]. The adsorption of Cr(VI) by disodium ethylene-diaminetetraacetate (EDTA-2Na)-modified GO/CS (EDTA/GO/CS) composite was evaluated; its adsorption capacity of 86.17 mg/g was relatively lower. However, the regeneration of the EDTA/GO/CS composite was feasible; the adsorbent exhibited a lowered capacity by 5% for Cr(VI) after seven times of reuse [166].

Tetraethylenepentamine (TEPA)-modified wheat straw (TEPA-WS) showed a sorption capacity of up to 454 mg/g for Cr(VI) due to the abundant grafted N- groups [167]. The grafting/polymerization of GMA onto bamboo hydrochar was performed, which was then aminated with diethylenetriamine and subsequently treated by hydrochloric acid [168]. Due to the bearing N^+^H_2_R group, it exhibited a superior adsorption capacity of 424.09 mg/g for Cr(VI). A chemical/physical modification of various adsorbents, using heteroatom-containing reagents, could significantly improve their ability to remove Cr(VI) from wastewater, providing some references for the construction and design of novel sorbents in the future.

Novel spongeous adsorbents showed a high performance and are cost-effective for the removal of various toxic metal ions from aqueous solutions. A polypyrrole (PPy)-modified natural core of corncob (PPy-NCC) was rationally fabricated for Cr(VI) removal from aqueous solution, and the adsorption capacity of PPy-NCC was several times higher than other bulk-style PPy composites due to its unique macroporous spongeous microstructure [169]. The co-pyrolysis and decomposition of cotton and polyester could obtain new biochar for the adsorption of Cr(VI), and the synergy effect contributed a lot to the efficient removal [170].

The positive surface charge of an adsorbent could improve its capacity and adsorption speed toward Cr(VI) [171]. The positively charged biochar made from pyrolyzed ramie residues could interact electrostatically with Cr(VI) ions, providing a low-cost and effective sorbent for Cr(VI) removal [172].

Dithiocarbamate (DTC)-modified starch derivatives, such as DTC functionalized starch (DTCS), DTC functionalized enzymolysis starch (DTCES) and DTC functionalized mesoporous starch (DTCMS), showed a significant adsorption performance for Cr(VI), with a sequence of DTCMS > DTCES > DTCS [173]. Recently, ACs prepared by industrial black liquor (BL) were activated with K_2_CO_3_ to obtain a higher surface area and larger pore volume, and relatively higher adsorption capacities of ~670 mg/g for Cr(VI) could be observed [174]. The combination of ZnO with biochar (ZBC) could produce novel adsorbents with an enhanced adsorption capacity, owing to the introduced electrostatic attraction, surface complexation and ion-exchange interactions [175].

### 2.11. Three-Dimensional (3D) Nanocomposites

Three-dimensional network structures possess larger surface areas, more abundant functional groups and highly exposed active sites and higher pore volumes, as well as good elasticity and excellent reusability. The favorable properties of 3D network structures make them become an active focus in the area of adsorption toward Cr species [176].

### 2.12. Hydrogels and Aerogels

Porous materials with size-adjustable pores and relatively higher surface areas could be used as efficient adsorbents. However, common adsorbents suffer from lower removal efficiencies due to their smaller surface area. To seek more effective adsorbents, recently, ice-segregation-induced self-assembly (ISISA), also called the unidirectional freeze-drying method (UFDM), was developed to produce porous aerogel materials with oriented structures. It is worth noting that the preparation procedure itself is very facile and environmentally friendly.

**Figure 6:**
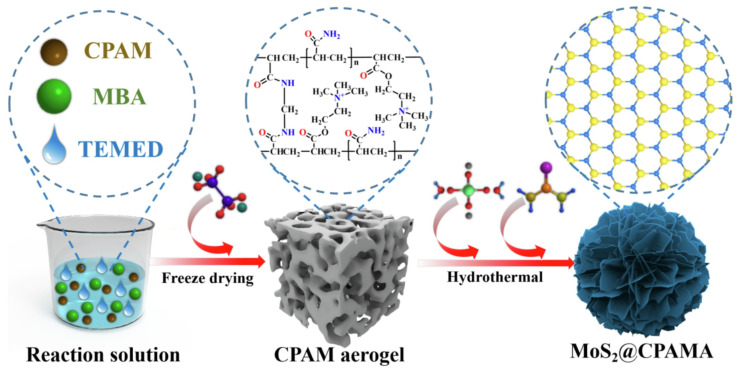
Preparation route of MoS_2_@CPAMA [177]. Copyright belongs to Elsevier B.V.

A N^+^(CH_3_)_3_-containing cationic polyacrylamide aerogel (CPAMA) was intercalated into MoS_2_ (MoS_2_@C-PAMA), and the active sites of MoS_2_ were well exposed for facilitating the adsorption and reduction of Cr(VI) (Figure 6). The high removal efficiency of 95% for Cr(VI) within 50 min and the maximum adsorption capacity of 800.0 mg/g demonstrated its robust removal ability [177]. A 3D honeycomb-like eco-friendly PEI/sodium alginate (SA) aerogel can be fabricated via a one-step freeze-drying procedure. Due to the excellent compressibility and plentiful active adsorption sites, the fast and highly efficient adsorption of Cr(VI) (678.67 mg/g) could be obtained [178,179]. Based on a similar principle, a design of functionalized cellulose-based aerogel beads (CGP) was performed, and the simultaneous and adsorption, reduction and elimination of Cr(VI) could be efficiently implemented [180]. If PEI and amino-functionalized Ti_3_C_2_T_x_ (a transition metal carbide, MXene) were incorporated into the SA aerogel matrix, the robust adsorption capacity of 538.97 mg/g toward Cr(VI) could be observed due to the abundant active groups and the in situ reduction ability of MXene [181]. For the carbon-based aerogels, the introduction of such multifunctional molecules also seems beneficial [182,183].

### 2.13. Frameworks

As potential emerging environmental materials, novel frameworks have received extensive attention in Cr removal. Due to the high nitrogen (N) content, covalent triazine frameworks (CTFs) were developed for the efficient adsorption of Cr(VI), and the strong ion-exchange interaction of quaternary N on CTF toward Cr(VI) was confirmed [184]. The metal–organic frameworks (MOFs), which are hybrid materials of organic ligands and metal ions or clusters, are another ideal material for Cr removal [185]. Due to the adjustable metal center, MOFs with an ordered porous structure could be further modified with specific ligands for more efficient and selective Cr(VI) removal [186]. AC modified with iron terephthalate (Fe-BDC), an Fe-based MOF, exhibited a high monolayer adsorption capacity of 100 mg/g and fast adsorption rate toward Cr(VI) [187]. A bimetallic organic framework, (Fe/Co)-BDC, was further developed, which exhibited a notable capacity of 588 mg/g toward Cr(VI) [188]. The pectin-hydrogel-crosslinked Fe-based MOFs by calcium chloride was developed, which a showed higher adsorption capacity toward Cr(VI) (825.97 mg/g), indicating that the rational introduction of multifunctional groups onto MOFs was necessary [189].

Zeolitic imidazolate frameworks (ZIFs), a subgroup of MOFs, possess more excellent physicochemical properties and are more advantageous for adsorption applications [190]. A Co/Zn-based ZIF adsorbent was developed for Cr(VI) removal, and a homogeneous monolayer adsorption with maximum capacities of 69.4 mg/g could be obtained [191]. ZIFs could be utilized for capacitive deionization (CDI) desalination to simultaneously remove both Cr(VI) ions and other salts, and the radial distribution function (RDF) and ion distribution were involved in the adsorption process, which provided a new application prospect for ZIFs [189].

**Table 1:** Adsorption of Cr species by different adsorbents.

Cr Species	Type of Adsorbent	Adsorption Conditions	Adsorption Capacities	Ref.
Cr(VI)	FeMnO_x_/MWCNTs	*C*_0_ = 50 mg/L; adsorbent dose: 1 g/L; contact time: 60 min; pH: 2.0	47.25 mg/g	[34]
Cr(III)	GO@CZ	*C*_0_ = 30 mg/L; adsorbent dose: 300 mg/L; contact time: 60 min; pH = 7.0	285.71 mg/g	[43]
Cr(VI)	Gr-Si-PPy	*C*_0_ = 100 mg/L; adsorbent dose: 400 mg/L; T = 25 °C; contact time: 60 min; pH: 2.0	429.2 mg/g	[47]
Cr(VI)	GO@SiO_2_@C@Ni-400	*C*_0_ = 20 mg/L; T = 25 °C; adsorbent dose: 0.15 g/L; pH = 3.0	299.20 mg/g	[50]
Cr(VI)	GO-NiFe LDH	*C*_0_ = 20 mg/L; T = 30 °C; contact time: 280 min; adsorbent dose: 80 mg/L	53.6 mg/g	[51]
Cr(VI)	F_e3_O_4_-GO	*C*_0_ = 600 mg/L; T = 25 °C; adsorbent dose: 125 mg/L; pH = 6.0	280.6 mg/g	[55]
Cr(VI)	TSGA	*C*_0_ = 50 mg/L; T = 25 °C; adsorbent dose: 800 mg/L; contact time: 40 min; pH = 2.0	100% removal	[60]
Cr(VI)	APTES-NPSi	*C*_0_ = 200 mg/L; T = 25 °C; adsorbent dose: 5 mg; contact time: 180 min; pH = 2.0	103.75 mg/g	[64]
Cr(VI)	MnFe-LDH/MnFe_2_O_3_@3DNF	*C*_0_ = 300 mg/L; T = 25 °C; contact time: 120 min; adsorbent dose: 5 mg; pH = 2.0	564.88 mg/g	[121]
Cr(VI)	3D porous CoFe_2_O_4_@SiO_2_-NH_2_	*C*_0_ = 150 mg/L; contact time: 600 min; adsorbent dose: 1 g/L; T = 25 °C; pH = 2.0	126.8 mg/g	[138]
Cr(VI)	TCMR	*C*_0_ = 150 mg/L; contact time: 360 min; adsorbent dose: 2 g/L; T = 25 °C; pH = 5.0	27.04 mg/g	[150]
Cr(VI)	Rice husk	*C*_0_ = 100 mg/L; contact time: 60 min; adsorbent dose: 50 g/L; T = 25 °C; pH = 5.0–6.0	30 mg/g	[158]
Cr(VI)	BCS and BCW	*C*_0_ = 320 mg/L; contact time: 24 h; adsorbent dose: 4 g/L; T = 25 °C; pH = 2.0	24.6 mg/g for BCS, 23.6 mg/g for BCW	[159]
Cr(VI)	SDBC	*C*_0_ = 100 mg/L; contact time: 24 h; adsorbent dose: 1 g/L; T = 25 °C; pH = 5.0	688~738 μmol/g	[161]
Cr(VI)	ZBC	*C*_0_ = 100 mg/L; contact time: 600 min; adsorbent dose: 4 g/L; T = 25 °C; pH = 1.0	33.87 mg/g	[175]
Cr(VI)	PEI/SA	*C*_0_ = 240 mg/L; contact time: 300 min; adsorbent dose: 400 mg/L; T = 25 °C; pH = 2.0	678.67 mg/g	[178]
Cr(VI)	CGP	*C*_0_ = 100 mg/L; contact time: 800 min; adsorbent dose: 2 g/L; T = 25 °C; pH = 2.0	386.40 mg/g	[180]
Cr(VI)	Fe-BDC	*C*_0_ = 50 mg/L; contact time: 60 min; adsorbent dose: 50 mg; T = 25 °C; pH = 5.5	100 mg/g	[187]
Cr(VI)	(Fe/Co)-BDC	*C*_0_ = 50 mg/L; contact time: 60 min; adsorbent dose: 50 mg; T = 20 °C; pH = 5.3	588 mg/g	[188]
Cr(VI)	Pectin-hydrogel-crosslinked Fe-based MOFs	*C*_0_ = 50 mg/L; contact time: 60 min; adsorbent dose: 20 mg; T = 50 °C; pH = 3.0	825.97 mg/g	[189]
Cr(VI)	Co/Zn-based ZIF	*C*_0_ = 15.0 mg/L; contact time: 30 min; adsorbent dose: 33 mg; T = 25 °C; pH = 6.5	69.4 mg/g	[191]
Cr(VI)	NH_2_-SBA-15	*C*_0_ = 25.0 mg/L; contact time: 4 h; adsorbent dose: 100 mg; T = 30 °C; pH = 2.0	Removal efficiency of 88%	[192]
Cr(VI)	*Ficus carica* bast fiber	*C*_0_ = 350.0 mg/L; contact time: 210 min; adsorbent dose: 0.5 g; T = 25 °C; pH = 3.0	19.68 mg/g	[193]
Cr(VI) and Cr(III)	Canadian peat and coconut fiber	*C*_0_ = 250.0 mg/L; contact time: 20 h; adsorbent dose: 1.0 g; T = 25 °C; pH = 1.5	19.21 mg/g for Cr(III) and 9.54 mg/g for Cr(VI), respectively	[194]

## 3. Novel Adsorption Technologies

Based on the basic principle of adsorption, novel technologies such as continuous fluidized bed process, membrane separation, capacitive deionization (CDI) and so on have also been developed and attracted more and more attention.

### 3.1. Continuous Fluidized Bed Process

The removal of Cr species from wastewater via the continuous fluidized bed process has shown a good application prospect. A circulated fluidized bed based on a non-hazardous residue, petroleum coke fly ash (FA), was used to remove Cr(VI). Due to the reduction of Cr(VI) to Cr(III) by FA and the neutralization effect, the efficient removal of Cr(VI) from acidic wastewaters could be carried out (eluting agent = acetic acid, liquid/solid ratio = 20 L/kg, agitation time = 18 h, pH = 4 and temperature = 25 °C) [195]. The use of an Intermittent Bubbling Fluidized Bed (IBFB) based on self-assembled modified *Pleurotus cornucopiae* material (SMPM) could remove Cr(VI) more efficiently (inner diameter = 0.04 m, height = 0.6 m, Cr(VI) volume = 500 mL, 100–500 mg/L, pH = 2–5 and temperature = 20–30 °C) [196].

### 3.2. Membrane Technology

Membrane separation was used as a cost-effective technology for the treatment of wastewater due to its relatively higher reutilization ratios. The removal capacity of a novel membrane made from polyaniline/poly(ethylene-co-vinyl alcohol) (PANI/EVOH) nanofiber composite toward Cr(VI) was 93.09 mg/g. In addition, the removal efficiency could be maintained up to 92.8% in the first 5 adsorption–desorption cycles and to 76.7% even after 25 cycles [197]. Catalytic membrane technology is highly efficient and feasible for wastewater remediation. The controllable immobilization of ultrasmall palladium nanoparticles (PdNPs) on the filter-paper-based botryoidal nanolignin channel (Pd@LNP/FP) membrane was performed, and the complete removal of Cr(VI) within 2.5 min via the synergetic filtration enrichment and catalysis reduction confirmed its high efficiency and feasibility [198].

### 3.3. Capacitive Deionization (CDI)

**Figure 7:**
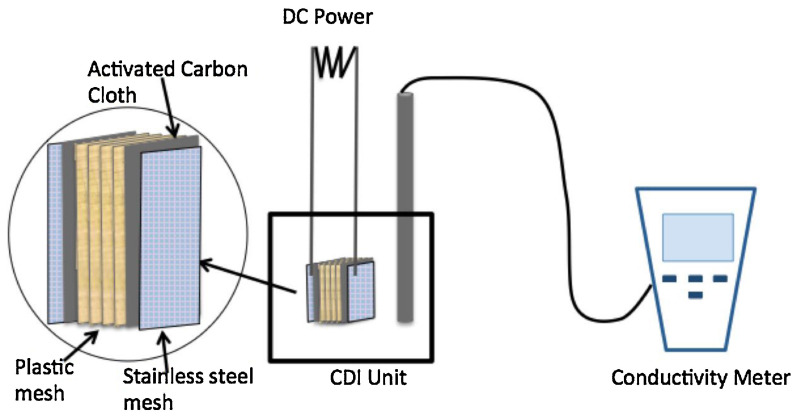
A schematic of CDI for metal removal [200]. Copyright belongs to Elsevier B.V.

The conventional adsorption method suffers from some problems, such as limited efficiencies, potential organic matter fouling, high cost or low regeneration rates. Capacitive deionization (CDI) has developed as a highly efficient and low-cost methodology for metal ions removal due to its lower life-cycle cost, higher recovery rate, lower energy consumption and more eco-friendly in use [199]. Recently, CDI technology has been preliminarily investigated for desalination treatment of Cr-contaminated water. The viability and preference of capacitive deionization (CDI) for removing Cr(III) were evaluated, and the removal efficiency was positively correlated with the applied voltage (Figure 7) [200].

An activated carbon (AC) electrode was fabricated and further used for the electro-sorption of Cr(VI) or low-concentration Cr(VI) (10 mg/L) with relative higher efficiency (97.1%) [201]. Acid-treated AC from rice husk was used as a CDI electrode for the electro-sorption of Cr(VI), and a relatively lower maximum capacity of 2.8316 mg/g was obtained [202]. Highly porous AC from a *Limonia acidissima* shell-based electrode contributed a lot to the membrane capacitive deionization (MCDI) of Cr(VI) with higher efficiency [203]. In the presence of bovine serum albumin (BSA), the amount for the adsorption of Cr(VI) by MCDI increased to 190.8 mg/g due to the stronger driving forces [204]. Taking advantage of synergistic effects, the combination of photocatalyst (MIL-53(Fe)) and CDI was performed, and an enhanced removal of total Cr with a high removal ratio (72.2%) could be achieved.

Recently, AC from Bael fruit shell (BS) with a macroporous and mesoporous structure exhibited a relatively larger specific surface area (617.72 m^2^/g). The removal of Cr(VI) by CDI with a BS-based electrode was performed, and an extremely high removal efficiency of ~100% at an applied potential of 15 V could be obtained due to its high adsorption capacity [205]. Obviously, the results are getting better and better. However, it is worth mentioning that the application of CDI in removing Cr ions is still in its infancy. More efforts and scientific research are needed to push the technology forward.

## 4. Mechanism Studies

### 4.1. Adsorbent–Adsorbate Interactions

The electrostatic (physical) and ionic (chemical) interactions would be involved in the Cr removal due to the higher aromatic structure of most carbon-based adsorbents [172]. For ACs, the removal of Cr(VI) might occur through intraparticle diffusion, electrostatic attraction and adsorption-coupled reduction [174]. The abundant carboxyl and hydroxyl groups on the oxidized carbons were found to be beneficial to the enhanced adsorption efficiencies [172]. Sorption only occurred at localized sites of PEI functionalized magnetic hydrochar, and the interaction between Cr(VI) and -NH_2_ could be confirmed [206].

Endowing the adsorbents with some unique properties, such as reduction and catalytic performance, would effectively improve the removal efficiencies. The chemical sorption between PANI/H-TNB and Cr(VI) occurred due to the combined effect of reduction of Cr(VI) to Cr(III), which contributed a lot to the higher adsorption efficiency [126].

Taking full advantage of the surface electrostatic adsorption, chelation, ion-exchanging and reduction effects, newly developed adsorbents would possess relatively higher adsorption capacities and a faster adsorption rate toward Cr species [178]. The enhanced removal efficiency for Cr(VI) by MCDI might be attributed to ion adsorption, redox reaction of Cr(VI) into Cr(III) and precipitation effects [204].

### 4.2. Analyses of Adsorption Process

To properly understand an adsorption process, the migration of adsorbate(s) from solutions onto adsorbents should be further clarified. In recent years, linear and nonlinear regression analyses of adsorption equilibrium data, including dynamics and isotherms, have been utilized to elucidate the adsorption process. To define the best fitting adsorption models that could quantify the distribution of adsorbates, analyze the adsorption process and verify the theoretical assumptions, various efforts have been made to elucidate the nature of the adsorbate–adsorbent interactions.

As shown in Table 2, almost all adsorption kinetics could be best fitted by the pseudo-first or pseudo-second order rate laws. If an adsorption is diffusion controlled, the process will follow the pseudo-first order sorption rate. Obviously, the pseudo-second order is the superior model, as it can represent many adsorption systems, thus indicating that the physicochemical interactions between the two phases, involving surface adsorption and chemisorption, dominated the rate-limiting step. Other kinetic models, such as the Elovich model, might describe some adsorption processes, indicating the adsorption behaviors concurred with the nature of chemical adsorption [206]. To construct more comprehensive equilibrium and dynamic models for predicting the competitive adsorption and the dynamic behaviors of coexisting metal ions, the adsorption of Ni(II), Co(II), Cr(VI) and P(V) by goethite was simulated by an equilibrium model, CD-MUSIC, and further analyzed by in situ ATR–FTIR spectroscopy [207]. The successful prediction of the binary competitive adsorption of Ni(II)-Co (II) and Cr(VI)-P(V) indicated that the developed model might be used as a robust and scalable solver to elucidate the adsorption procedure.

The adsorption equilibrium could be illustrated by the Freundlich, Langmuir, Tempkin and Redlich–Peterson isotherms. Among them, the Langmuir isotherm was the most widely suitable to simulate the monolayer adsorption of Cr species onto the homogenous adsorbents’ surfaces. The Freundlich isotherm could be applicable to an adsorption process that occurs on the heterogonous surface, defining the surface heterogeneity and the exponential distribution of active sites and their energies. Obviously, most adsorption processes obey the Langmuir model, thus confirming the homogenous surfaces obtained due to the well-developed fabrication technologies and the monolayer adsorption of Cr species on the adsorbents’ surface with specific functional groups.

Unfortunately, only most-used empirical models (such as the Elovich, intraparticle diffusion, pseudo-first-order and pseudo-second-order kinetic models, and Langmuir and Freundlich isotherm models) were used to understand the Cr species adsorption processes. Other theoretical adsorption models (kinetic models such as Langmuir rate equation, pseudo-n-th-order (PNO) equation, mixed 1,2-order equation (MOE), fractal-like kinetic models, Vermeulen and fractal-like Vermeulen models, Weber–Morris model, Bangham–Sever model, Furusawa–Smith model, Haerifar–Azizian model and other empirical models; isotherm models such as linear models (Redlichepeterson (ReP) isotherm, Sips isotherm model and Toth isotherm model); models based on Polanyi’s potential theory (Dubinin–Radushkevich (D–R) model); the Dubinin–Astakhov (D–A) model; chemical adsorption models (Volmer isotherm model); and physical adsorption models (BET model, Aranovich model and ion-exchange isotherm model)) are expected to propose the adsorption mechanisms in the future.

**Table 2:** Adsorption isotherm and kinetic studies of Cr removal from aqueous solution.

Type of Cr	Type of Adsorbent	Applicable Kinetic Model	Applicable Isotherm Model	Reference
Cr(VI)	MCMs	The pseudo-second-order model	The Langmuir model	[22]
Cr(VI)	FeMnO_x_/MWCNTs	The pseudo-second-order model	The Langmuir model	[34]
Cr(VI)	Gr-Si-PPy	The pseudo-second-order model	The Langmuir model	[47]
Cr(VI)	nZVI-MSC	The pseudo-first-order model	Not provided	[61]
Cr(VI)	nFeOOH@D001	The pseudo-second-order model	The Langmuir model	[73]
Cr(VI)	FeBC	The intra-particle diffusion model	Not provided	[79]
Cr(VI)	Fe_2_(SO_4_)_3_@Egeria najas based biochar	The pseudo-second-order model	The Langmuir model	[86]
Cr(VI)	PPy-MSFA	The pseudo-second-order model	The Langmuir model	[98]
Cr(VI)	SnZVI@EPS	The pseudo-second-order model	Not provided	[107]
Cr(VI)	Zn/Al-LDHs	The pseudo-second-order model	The Langmuir model	[116]
Cr(VI)	PANI-Mg/Al LDHs	The pseudo-second-order model	The Langmuir model	[118]
Cr(VI)	Crayfish shell biochar–Fe composite	The pseudo-second-order model	The Langmuir model	[134]
Cr(VI)	EDTA/GO/CS	The pseudo-second-order model	The Freundlich model	[166]
Cr(VI)	ZBC	The pseudo-second-order model	The Langmuir model	[175]
Cr(VI)	PEI-modified Juncus effuses	The pseudo-second-order model	The Freundlich model	[176]
Cr(VI)	Co/Zn based ZIF	Not provided	The Langmuir model	[191]
Cr(VI)	PANI/EVOH	The pseudo-second-order model	The Freundlich model	[197]
Cr(VI)	Pd@LNP/FP	The pseudo-first-order model	Not provided	[198]
Cr(VI)	PEI functionalized magnetic hydrochar	The Elovich model	The Freundlich model	[206]
Cr(VI)	CVN	The pseudo-second-order model	The Langmuir model	[208]

## 5. Conclusions and Future Perspectives

As an efficient method for wastewater treatment with relatively lower cost, better flexibility and simplicity of operation, higher ease of design and sensitivity to various contaminants, adsorption has attracted continuous attention in recent years. The current adsorbents for the decontamination of Cr species from wastewater still exhibit some limitations, such as high cost, low reproducibility, potential adverse effects and the possibility of producing secondary wastes. The development of reusable adsorbents will be helpful to correct this deficiency.

As discussed before, defect engineering could greatly enhance the removal efficiency of an adsorbent; further investigations could be performed to introduce defects, rather than compositing with other substances, which might be more eco-friendly, sustainable and practical.

Adsorbents with multifunctional properties, such as catalytic characteristics, are expected. The reduction of Cr(VI) to Cr(III) would greatly decrease the toxicity of Cr-species-based contaminations.

Unfortunately, only most-used empirical models (such as the Elovich, the intraparticle diffusion, the pseudo-first-order and pseudo-second-order kinetic models, and the Langmuir and Freundlich isotherm models) were used to understand the Cr species adsorption processes. Other theoretical adsorption models are expected to propose adsorption mechanisms in the future.

New technologies need to be developed for the more efficient and facile removal of Cr species. Membrane technology, CDI and the continuous fluidized bed process have shown excellent decontamination properties, and the environmentally friendly features, economic effectiveness and recycling features undoubtedly guarantee their wide and practical applications in the future. Some general limitations associated with the adsorbent in a powder media include surface area loss, process control and production time/cost. To control the packing density and pressure drop of the media while immobilizing the adsorbent, future research on adsorption technologies should primarily focus on the practical applications of fixed columns with the packing adsorbent in wastewater treatment plants.

## Data Availability

Not applicable.

## Abbreviations

3D, three-dimensional; Ag-ACE, nanosilver-assisted chemical etching; ASGO, amino-modified graphene oxide; α-FeOOH, goethite; β-FeOOH, akageneite; γ-FeOOH, lepidocrocite; δ-FeOOH feroxyhyte; AC-NH_2_, quaternary amine-anchored AC; ACs, active carbons; BC, biochar; BC-SnZVI, sulfide-modified nanoscale zero-valent iron (SnZVI) anchored BC; BL, black liquor; BLN, pulping black liquor lignin; CDI, capacitive deionization; CG-RS, coal gangue–rape straw biochar; CGP, cellulose-based aerogel beads; CHS, sulfonated carbon hemisphere; CMC, carboxymethyl cellulose; CMS, carbon microsphere; CNFs, carbon nano fibers; CNTs, carbon nanotubes; CPAMA, cationic polyacrylamide aerogel; Cr, chromium; Cr(II), chromous ion; Cr(III), chromic ion; Cr(VI) or or CrO_4_^2−^, chromate ion; (Cr_2_O_3_), Cr(III) oxide; Cr(OH)_3_, Cr(III) hydroxide; Cr_2_O_7_^2−^, dichromate ion; CTFs, covalent triazine frameworks; DETA, diethylenetriamine; ELN, enzymolytic lignin; EPS, extracellular polymeric substance; FeBC, Fe-modified BC; FeOOH, iron oxyhydroxide; FeS, ferrous sulfide; FeSO_4_, iron sulphate; FS, SPORL pretreatment spent liquor; GMA, glycidylmethacrylate; Gr, graphene; GO, graphene oxide; HA, humic acid; HFO, hydrated iron oxide; KH-560, 3-glycidoxypropyltrimethoxysilane; L-cys, L-cysteine; LDH, layered double hydroxide; MCMSs, magnetic carbon microspheres; MOFs, metal–organic frameworks; MPCMS, mesoporous carbon microsphere; MPMs, magnetic polymer microspheres; MSC, mesoporous silica–carbon; NPs, nanoparticles; nZVI, nanoscale zero-valent iron; NH_2_-SBA-15, amino-functionalized SBA-15; PA, polyamine; PAH, poly(allylamine hydrochloride); PDMDAAC, poly(dimethyl diallyl ammonium chloride); PEI, polyethyleneimine; PPy, polypyrrole; PRBs, permeable reactive barriers; PS, polystyrene; PVC, polyvinyl chloride; PVP, polyvinylpyrrolidone; Q_m_, adsorption capacity; *R*, removal percentage; RDF, radial distribution function; rGO, reduced graphene oxide; SA, sodium alginate; SnZVI, sulfidated nanoscale zero-valent iron; SnZVI@EPS, sulfidated nanoscale zero-valent iron@EPS; SSA, specific surface area; TCMR, traditional Chinese medicine residual; TEPA, tetraethylenepentamine; TETA, triethylentetramine; TSGA, TiO_2_-Zn_x_Cd_1-x_S rGO aerogel; ZBC, ZnO modified biochar; ZIFs, Zeolitic imidazolate frameworks.
